# Parasites and allergy: Observations from Africa

**DOI:** 10.1111/pim.12589

**Published:** 2018-10-21

**Authors:** Harriet Mpairwe, Abena S. Amoah

**Affiliations:** ^1^ Medical Research Council/Uganda Virus Research Institute and London School of Hygiene and Tropical Medicine Uganda Research Unit Entebbe Uganda; ^2^ Department of Parasitology Leiden University Medical Center Leiden The Netherlands

**Keywords:** Africa, allergy/atopy, Helminths, immune mechanisms, immunoepidemiology, immunoglobulin, parasite, tools and techniques

## Abstract

Population studies from the African continent have observed a marked increase in the prevalence of allergy‐related diseases over the past few decades, but the cause of this rise is not fully understood. The most investigated potential risk factor has been the relationship between exposure to helminths and allergy‐related outcomes. Immunologically, parallels exist between responses to helminths and to allergens as both are associated with elevated levels of immunoglobulin E, increased numbers of T helper 2 cells and other immune cells. However, epidemiological studies from the African continent have found inconsistent results. In this review, observations from population studies carried out in Africa over the last decade that focus on the relationship between helminth infections and allergy‐related outcomes are examined. How these findings advance our understanding of the complex interactions between helminths and allergies at the population level is also explored as well as some of the underlying immune mechanisms involved. This knowledge is important for better diagnosis, treatment and prevention of allergy‐related diseases and has wider global significance.

## 1 INTRODUCTION

The global burden of helminth infections remains high with an estimated 1.5 billion people worldwide chronically infected with at least one soil‐transmitted helminth^1^ and 240 million having the waterborne helminth disease schistosomiasis.^2^ It is also estimated that 92% of those with schistosomiasis live in sub‐Saharan Africa.^3^

Helminth infections are strongly linked to poverty, poor hygiene and inadequate sanitation and are more common in rural locations.^4^, ^5^ Reduced exposure to helminths is thought to be one of the factors driving the rise in the incidence of allergic disease worldwide.^6^ Increases in the prevalence of allergy‐related diseases over time have also been seen on the African continent especially among children,^7^, ^8^ particularly in urban compared to rural areas.^8^, ^9^, ^10^, ^11^, ^12^, ^13^, ^14^ Aside from helminths, other factors have been linked to this rise including urbanization, less exposure to childhood pathogens in general and lifestyle changes.^15^

Helminths are of specific interest to investigations on allergy‐related diseases due to the immunological parallels between these two conditions. Both are associated with T helper 2 (Th2) cell induction, high levels of immunoglobulin E (IgE) and the involvement of immune cells such as mast cells, basophils and eosinophils.^16^, ^17^ While immunological parallels exist, the resultant clinical outcomes are dissimilar and chronic helminth infections can induce an immune regulatory network in the host resulting in an anti‐inflammatory environment as well as general T‐cell hyporesponsiveness.^18^ Although some epidemiological studies conducted in helminth‐endemic countries have reported an inverse association between helminths and allergy‐related outcomes, other investigations have observed either no association or even a positive association. In this review, we highlight findings from population studies conducted in Africa that explore the relationship between helminths and allergy‐related outcomes with a particular emphasis on studies conducted within the past decade.

We identified relevant publications through searches conducted in PubMed using keywords related to “allergy” or “hypersensitivity” in combination with “helminths,” “parasites,” or “worms” and “Africa (South of the Sahara and Northern Africa).” Our search was restricted to human studies reported in the English language that were published between January 2008 and March 2018. The search was limited to the last decade to highlight new and recent findings in the literature given that older observations have been covered extensively elsewhere.^19^, ^20^, ^21^, ^22^, ^23^

In this review, we first explore findings on helminths and reported/clinical allergy outcomes from epidemiological studies conducted in the past decade. Our reported/clinical allergy outcomes included asthma, wheeze, exercise‐induced bronchospasm, rhinitis, conjunctivitis, eczema, reported adverse reactions to peanut and skin prick test (SPT) reactivity to allergens. We then examine population study findings related to immune mechanisms including helminths and IgE sensitization, cellular immune mechanisms and other pathways through which helminths may protect or increase susceptibility to allergy‐related diseases.

## 2 HELMINTHS AND REPORTED OR DIAGNOSED ALLERGY‐RELATED OUTCOMES

### 2.1 General characteristics of studies

**Figure 1:**
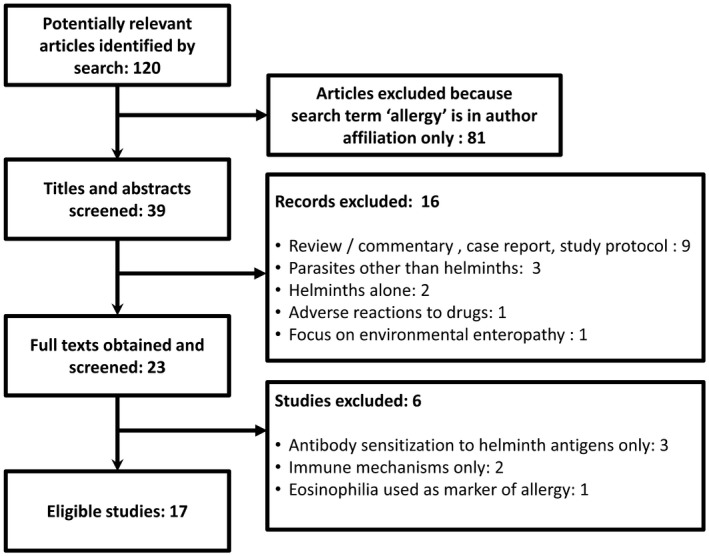
Flow diagram showing selection of publications for inclusion in review of population studies on helminths and allergy‐related outcomes in Africa

Epidemiological studies that investigated the relationship between helminths and allergy‐related outcomes (self‐reported, doctor‐diagnosed or SPT), between January 2008 and March 2018, are shown in Table 1. A flow diagram showing the selection of publications for inclusion is displayed in Figure 1. In general, studies were (a) mostly observational, as either cross‐sectional or case‐control studies, (b) conducted among school‐age children or adults, (c) conducted in either urban or rural areas or both and (d) one study was an intervention of anthelmintic treatment in pregnancy with subsequent follow‐up of the offspring into childhood as part of a birth cohort.

**Table 1:** Summary of articles investigating associations between helminths and allergy‐related diseases outcomes in Africa from January 2008 to March 2018

Article	Sample size (N) & Study design	Age (years)	Country & Setting	Worm burden (%)	Allergy‐related outcomes	Effect size [OR/HR (CI), *P*‐value]
Hartgers, et al^24^	123 Cross‐sectional	5‐14	Ghana Rural	Any helminth—52% *S. haematobium*—38% Hookworm—24%	SPT in *S. haematobium* uninfected —19%, SPT in *S. haematobium* infected —11%	*S. haematobium* ↔SPT 0.26 (0.07‐1.00), *P* = 0.05
Calvert, and Burney, 11	773 Case‐control	8‐12	South Africa Rural & Urban	*Ascaris*—61% *T. trichiura*—33%	EIB in rural—8.7% EIB in urban—14.9% SPT with EIB —20.7% SPT without EIB —3.7%	*Ascaris* ↔EIB 1.87 (1.19‐2.95), *P* = 0.009 *T. trichiura* ↔EIB 0.99 (0.74‐1.35), *P* = 0.99 *Ascaris* ↔SPT 0.63 (0.42‐0.94), *P* = 0.03
Amberbir, et al^25^	878 Cross‐sectional	3	Ethiopia Rural & Urban	Hookworm—4.9% *A. lumbricoides*—4.3% Any geo‐helminths—8.5%	Wheeze—9% Eczema—6.3% Hay fever—5% Any SPT—8.7%	Any geo‐helminths ↔ Wheeze [0.74 (0.29‐1.90), *P* = 0.53] Eczema [0.39 (0.09‐1.63), *P* = 0.19] Hay fever [0.49 (0.12‐2.09), *P* = 0.33] SPT [1.25 (0.57‐2.73), *P* = 0.58]
Ige, et al^26^	110 Case‐control	Adults, mean = 38	Nigeria Rural & Urban	*Taenia solium*—11% (asthmatics) vs 13% (controls) *A. lumbricoides*—11% (asthmatics) vs 9% (controls)	55 asthmatics & 55 controls	Not significant (values not reported)
Larbi, et al^27^	1482 Cross‐sectional	6‐15	Ghana Rural & Urban	*S. haematobium*—2.7% urban, 10% rural Hookworm—2% urban, 12% rural *Ascaris spp*—0% urban, 14.5% rural *Trichuris spp*—0.3% urban, 2.5% rural	SPT in urban—17.8% SPT in rural—25%	Single helminths – Not significant
Mpairwe, et al^28^	2507 RCT^a^	0‐1	Uganda Peri‐urban	Mother's worms in pregnancy: Hookworm—44% *S. mansoni*—18.3%	Eczema in first year of life—rate 10.4/100 PYFU Reported recurrent wheeze at 1 y—9%	Albendazole in pregnancy→eczema (physician‐diagnosed, 0‐1 y) 1.82 (1.26‐2.64), *P* = 0.002 Praziquantel in pregnancy (if mother had *S. mansoni*) →eczema (physician‐diagnosed 0‐1 y) 2.65 (1.16‐6.08), not significant if mother had no *S. mansoni*; interaction *P* = 0.02. Albendazole in pregnancy→reported wheeze (at 1 y) 1.58 (1.13‐2.22), *P* = 0.008
Smedt, et al^29^	3041 Cross‐sectional	7‐14	Rwanda Rural & Urban	Any of 10 helminth species (eggs) in stool—23.1%	Vernal Keratoconjunctivitis—4%	Any helminths↔vernal keratoconjunctivitis 1.0 (0.6‐1.7), *P* = 0.95
Stevens, et al^30^	198 Case‐control	6‐16	Ghana Rural & Urban	Only 4 had any helminths	99 asthmatics & 99 controls	Not significant (values not reported)
Rujeni, et al^31^	672 Cross‐sectional	1‐86	Zimbabwe Rural	*S. haematobium*—52.9% & 8.6% in high & low transmission areas	SPT—17.7%	SPT size inversely related to *S. haematobium* intensity, in high transmission area only r = ‐0.101, *P* = 0.037
Amare, et al^32^	405 Cross‐sectional	Mean 12.09 ± 2.54	Ethiopia Town	Any helminth—22.7%, *A. lumbricoides*—48%, *Hymenolepis nana*—28%, Hookworm—9%, *T. trichiura*—6.6%	History of any reported allergy—8%	Associations between helminths reported as not significant (actual numbers not shown)
Amoah, et al^33^	1604 Cross‐sectional	5‐16	Ghana Rural & Urban	Any intestinal helminths—18% *S. haematobium*—7%	Adverse reactions to peanuts—1.5% Peanut SPT—2%	Any intestinal helminths ↔reported adverse reactions to peanut 0.35 (0.08‐1.56), *P* = 0.17 Any intestinal helminths ↔peanut SPT 0.69 (0.17‐2.84), *P* = 0.61 *S. haematobium* ↔ reported adverse reactions to peanut 0.65 (0.08‐4.95), *P* = 0.67 *S. haematobium* ↔peanut SPT 0.41 (0.05‐3.42), *P* = 0.41
Oluwole, et al^34^	170 Case‐control	13‐14	Nigeria Rural & Urban	*A. lumbricoides*—17% asthmatics, 13% controls Hookworm—5% asthmatics, 4% controls	SPT—73% asthmatics, 60% controls	No statistically significant associations reported (actual values not shown)
Mpairwe, et al^35^	2507 pregnant women, 2345 live births Birth cohort^b^	0‐5	Uganda Peri‐urban	Mother's helminths in pregnancy: Hookworm—45%, *Mansonella perstans*—21%, *S. mansoni*—18%, *Strongyloides stercoralis*—12%, *T. trichiura*—9% Children's worms in first 5 y: *T. trichiura*—21%, *A. lumbricoides*—11%, *S. mansoni*—7%, Hookworm—6%	Doctor‐diagnosed eczema‐rate in first 5 y—4.68/100 PYFU	Mother's hookworm→eczema 0‐5 y 0.71 (0.51‐0.99), *P* = 0.04 Mother's hookworm modified effects on other known eczema risk factors Mother's other helminths ‐not significant Childhood *T. trichiura* ↔eczema 0‐5 y 0.35 (0.18‐0.67), *P* = 0.002 Childhood hookworm ↔eczema 0‐5 y 0.33 (0.11‐1.02), *P* = 0.05 Other childhood helminths‐not significant
Obeng, et al^36^	1385 Cross‐sectional	5‐16	Ghana Rural & Urban	Hookworm—9.9% *Schistosoma* spp—9.5% *Ascaris* spp—6.2% *Trichuris* spp—1.9% Any helminths—23.1%	Reported asthma—8.2% Wheeze—7.9% Any SPT—18%	For wheeze and asthma, no significant associations were seen with single or combined helminth infections *Schistosoma* spp ↔mite SPT 0.15 (0.05‐0.41), *P* < 0.0001 *Schistosoma* spp ↔cockroach SPT 0.49 (0.18‐1.29), *P* = 0.15 *Trichuris* spp ↔cockroach SPT 3.73 (1.22‐11.41), 0.02; but no association with mite SPT No significant association between all other helminths and SPT
Pinot de Moira, et al^37^	240 Cross‐sectional	7‐16	Uganda Rural (Fishing village)	*S. mansoni*—93.8% Hookworm—80.4% Other helminths—37%	Wheeze—8.2% Dust mite SPT—4.2%	Hookworm ↔wheeze 0.29 (0.10‐0.87), *P* = 0.03 *S. mansoni* infection intensity ↔wheeze 1.05 (0.82‐1.34) Helminths ↔SPT reported as not significant (data not shown)
Webb, et al^38^	2316 Cross‐sectional survey	Median 24, IQR 8.32	Uganda Rural (Fishing village islands)	*S. mansoni* (KK)—51%; (CCA)—72% Hookworm (PCR)—22% *S. stercoralis* (PCR)—12%; *T. trichiura*—10%; *A. lumbricoides*—1.2%	Wheeze <5 y—2% Wheeze ≥5 y—5% Any SPT—19%	*A. lumbricoides* ↔wheeze 6.36 (1.10‐36.63), *P* = 0.04 All other helminths—not significantly associated with wheeze *T. trichiura* ↔SPT 2.08 (1.38‐3.15), *P* = 0.001 All other helminths—not associated with SPT
Namara, et al^39^	1188 RCT (& birth cohort)	9	Uganda Peri‐urban	Mother's infection during pregnancy: Hookworm—42.6%, *S. mansoni*—19.3% Children's infections at 9 y: *S. mansoni*—11%, *T. trichiura*—4%, *A. lumbricoides*—1%	Wheeze—3.7% Doctor‐diagnosed eczema—2.3% Reported eczema—3.4% Doctor‐diagnosed asthma—1.2% Any SPT—25%	Maternal albendazole in pregnancy→wheeze at 9 y—0.70 (0.31‐1.57); SPT at 9 y—0.96 (0.68‐1.37) Maternal praziquantel in pregnancy→wheeze at 9 y—1.53 (0.69‐3.43); SPT—1.13 (0.79‐1.61) Childhood albendazole→wheeze at 9 y—1.01 (0.01 (0.46‐2.23); SPT at age 9 y—1.00 (0.71‐1.43)

OR, odds ratio; HR, hazard ratio; CI, confidence interval; SPT, skin prick test positive for allergic sensitization; EIB, exercise‐induced bronchospasm; RCT, randomized controlled trial; PYFU, person‐years of follow‐up; KK, Kato‐Katz method; CCA, circulating cathodic antigen; PCR, polymerase chain reaction

↔ the two variables tested for association in case‐control or cross‐sectional studies.

→ the two variables tested for association in clinical trial or birth cohort.

a RCT albendazole vs placebo and praziquantel vs placebo in pregnancy as 2 × 2 factorial design, followed by albendazole vs placebo in childhood 15 months to 5 years.

b Birth cohort following the RCT above.

### 2.2 Prevalence of helminths and allergy‐related outcomes

As shown in Table 1, studies conducted across the African continent show a wide range for the prevalence of helminth infections with some investigations having high burdens (>50%),^11^, ^31^, ^37^, ^38^ while others had low percentages (<10%)^25^, ^27^, ^30^, ^36^ according to WHO classification.^40^ Although the vast majority of investigations used the Kato‐Katz method for the detection of soil‐transmitted helminths and *Schistosoma mansoni*, and urine filtration for the detection of *S. haematobium*, a few studies used other methods such as formol‐ether concentration. In a few studies, methods used were not specified. The type of helminth infections among participants varied from study to study, but the most common helminths were *Schistosoma*, hookworm, *Ascaris* and *Trichuris* species. The most commonly studied allergy‐related outcomes were reported wheeze, eczema and asthma, all with a prevalence of less than 10%. The percentage of positive SPT responses varied from 2%^33^ to 73%.^34^ Generally, food allergens such as peanut elicited fewer SPT responses compared to environmental allergens such as house dust mite and cockroach.

### 2.3 Association between helminths and allergy‐related outcomes from observational studies

Information on associations between helminth infections and allergy‐related outcomes is summarized in Table 2. Although this is not a meta‐analysis, the total numbers of participants in the various studies are shown. Two of the three studies that showed a positive association were of *Ascaris* and respiratory symptoms of wheeze^38^ or exercise‐induced bronchospasms,^11^ and the third between *Trichuris* and SPT.^36^, ^38^ Although migration of *Ascaris* larva through the lungs is mostly asymptomatic, on rare occasions this may be associated with respiratory symptoms such as wheezing, dyspnoea and bronchospasm.^41^ Among rural and urban South African children, Calvert and Burney observed a positive association between *A. lumbricoides* infection and exercise‐induced bronchospasms, but an inverse association between *Ascaris* and SPT positivity,^11^ suggesting that the underlying mechanisms for SPT and bronchospasm are different.

**Table 2:** Summary of comparisons made for the association between helminths and allergy‐related diseases outcomes by studies conducted in Africa between 2008 and March 2018

Direction of effect	Number of published studies	Total number of participants in the given studies	Specific examples of comparisons made
Positive	3	4474	*A. lumbricoides* ↔EIB; *A. lumbricoides* ↔wheeze; *T. trichiura* ↔SPT (×2)
Inverse	6	5700	*Schistosoma* spp ↔SPT (×4); *A. lumbricoides* ↔SPT; Hookworm ↔ wheeze; Hookworm ↔ eczema; *T. trichiura* ↔eczema; Maternal hookworm in pregnancy ↔ eczema 0‐5 y
No statistically significant association reported by the whole study	8	7888	A wide range of combinations
Both statistically significant and insignificant results reported by the same study	4	6448	A wide range of combinations
Positive (anthelmintic treatment)	1	2507	Albendazole in pregnancy→eczema in infancy Albendazole in pregnancy→wheeze in infancy Praziquantel in pregnancy →eczema in infancy
No association (anthelmintic treatment)	1	1188	Albendazole in pregnancy→wheeze & SPT at age 9 y Praziquantel in pregnancy→wheeze & SPT at age 9 y Albendazole in early childhood→wheeze & SPT at 9 y

↔ The two variables tested for association in observational studies → The two variables tested for association in clinical trials

Of the six studies that showed an inverse association between helminths and allergy‐related outcomes (in Table 2), five of them were conducted in areas with a high proportion of individuals infected with helminths,^11^, ^24^, ^31^, ^35^, ^37^ while the sixth study was conducted in an area with low proportion of individuals infected with helminths.^36^ It is also important to note that although a study conducted among island communities in Lake Victoria where the proportion of individuals with helminth infections was high, no inverse associations with allergy‐related outcomes were reported.^38^

Tables 1 and 2 show that a large proportion of comparisons found no association between helminth infections and allergy‐related outcomes. There are several possible explanations for this observation. First, for most of these studies that showed no association, the prevalence of clinical allergy‐related outcomes was low,^25^, ^29^, ^32^, ^33^, ^38^, ^39^ and/or the prevalence of helminths was low, particularly in urban areas.^25^, ^27^ This may have resulted in wide confidence intervals and thus reduced power to detect a statistically significant difference. Some studies had small sample sizes and are likely to have been underpowered.^26^, ^30^, ^34^ Although the prevalence of SPT was generally higher in many investigations, its association with helminths was not consistent for reasons that are not well understood.

Secondly, misclassification of helminth status may have occurred since many of these studies relied on the examination of a single parasitological sample using microscopy, which is not very sensitive.^42^ Misclassification of allergy‐related outcomes may have occurred since most studies relied on self‐reported allergy‐related outcomes from a questionnaire rather than doctor‐diagnosed outcomes. Aside from questionnaires being a problem in settings where clinical diagnosis and translation of questionnaires into the local languages are a challenge, there may have been an over‐estimation or under‐estimation of self‐perceived allergy.^43^

Lastly, there is evidence that the risk of allergy‐related outcomes is established early in life,^44^ yet most investigations shown in Table 1 were conducted among school‐age children or adults. Therefore, it is possible that helminths at this point in life may have a limited role in modifying the risk or the clinical manifestation of allergy‐related outcomes. If this is the case, then investigating the possible role of helminths in early life (in utero and first few years) would be important.

### 2.4 Association between helminths and allergy‐related outcomes from anthelmintic intervention studies

At the time of this review, only one study in Africa had investigated the possible role of exposure to helminths in utero and early life on allergy‐related disease risk. This study, conducted in Uganda, was a randomized placebo‐controlled trial of albendazole vs placebo and praziquantel vs placebo (2 × 2 factorial design) in pregnancy, followed by albendazole vs placebo for the offspring between 15 months to 5 years of age.^45^ This investigation showed that anthelmintic treatment during pregnancy resulted in increased risk of eczema in infancy^28^ in the first 5 years^46^ and that maternal hookworm during pregnancy was associated with reduced risk of eczema in infancy and in the first 5 years of life.^35^ Albendazole treatment in early childhood was not associated with allergy‐related outcomes.^35^, ^46^ However, the effects of anthelmintic treatment in pregnancy on clinical asthma could not be measured due to the small number of children who developed asthma in this cohort.^39^ This birth cohort also showed that the children's own helminth infections in early life (hookworm and *Trichuris*) were associated with a reduced risk of eczema in later childhood.^35^ Furthermore, dust mite‐specific IgE in childhood was strongly associated with eczema if the mother had no hookworm [adjusted HR (95% CI), *P*‐value: 2.72 (1.11‐6.63), *P* = 0.03] but not if the mother had hookworm during pregnancy [0.41 (0.10‐1.69), *P* = 0.22; interaction *P*‐value = 0.03].^35^ This effect modification was significant for cockroach IgE and other known risk factors for eczema such as mother's history of eczema.^35^

## 3 HELMINTHS AND ALLERGY‐RELATED OUTCOMES: IMMUNE MECHANISMS IN POPULATION STUDIES

### 3.1 Dissociation between allergen‐specific IgE and SPT reactivity in Africa

Observations from Africa have shown that allergen‐specific IgE and SPT are not as strongly associated with allergy‐related outcomes as seen in high‐income countries and that this association is even weaker in rural compared to urban areas. For example, a study in Ghana found that the association between mite‐specific IgE and mite SPT positivity was strongest among individuals from an urban high socioeconomic status (SES) background [aOR (95% CI): 15.58 (7.05‐34.43)], followed by urban low SES participants [10.44 (5.60‐19.47)], and least among rural individuals [5.43 (3.83‐7.69); test for heterogeneity *P* = 0.007].^36^ The reasons for this dissociation are not fully understood, but there is evidence to suggest that helminths may play a role in IgE sensitization.

### 3.2 Helminth‐induced IgE cross‐reactivity

The role of helminths in stimulating the production of IgE antibodies was first postulated in a study published in the late 1960s in which a group of Ethiopian pre‐school children infected with the helminth *A. lumbricoides* were found to have 28 times higher levels of total IgE compared to Swedish children of the same age.^47^ Since then, elevated levels of allergen‐specific IgE associated with helminth infections that do not translate into allergy symptoms have been seen in numerous studies from Africa.^33^, ^36^, ^48^ This may partly be due to the phenomenon of IgE cross‐reactivity in which antibodies directed against one epitope recognize similar epitopes in homologous molecules.^49^ Research studies over the past few decades have linked two kinds of cross‐reactivity to allergens; cross‐reactivity due to proteins and cross‐reactivity due to the carbohydrate structures on glycoproteins known as cross‐reactive carbohydrate determinants (CCDs).

With regard to helminths and protein IgE cross‐reactivity, a number of allergens from invertebrate sources such as house dust mite, shrimp and cockroach have been shown to cross‐react with helminth antigens.^50^, ^51^ Proteins implicated include tropomyosin,^52^, ^53^, ^54^, ^55^ glutathione S‐transferase (GST)^56^, ^57^ and paramyosin.^58^

Although there are very few studies examining IgE cross‐reactivity between proteins from helminths and homologues from invertebrate allergen sources in African individuals, cross‐reactivity may explain some observations made in African studies in the past decade. For example, Levin et al observed a positive association between specific IgE to *Ascaris* antigen and SPT reactivity to aeroallergens among adolescents in South Africa.^59^ The authors postulated that cross‐reactivity between *Ascaris* proteins such as tropomyosin or GST and their corresponding homologues in house dust mite and cockroach may explain the positive SPTs. Additionally, cross‐reactivity between the filarial nematode *Onchocerca volvulus* tropomyosin (OvTrop) and house dust mite tropomyosin (Der p 10) has been demonstrated in vitro^53^ although populations studies are yet to be conducted linking onchocerciasis with elevated levels of specific IgE to house dust mite.

The sugar components of insect and plant glycoproteins known as CCDs are central to IgE carbohydrate cross‐reactivity.^60^ N‐linked glycans containing core α‐1,3‐fucose and β‐1,2‐linked core xylose are the most characterized motifs related to this cross‐reactivity.^61^ IgE antibodies against CCDs were first reported in the early 1980s,^62^ and their role in inducing high levels of IgE against peanut extract without peanut allergy symptoms was observed in the Netherlands.^63^

In Africa, a role for helminths in cross‐reactivity involving carbohydrates was demonstrated by an investigation conducted among schoolchildren in Ghana in which 18% were peanut IgE‐sensitized, but 92% of those sensitized were peanut SPT negative.^33^ Additionally, current *S. haematobium* infection was positively associated with peanut IgE sensitization and a strong correlation was observed between IgE to CCDs and IgE to whole peanut extract. In a subset of children in that study, inhibition assays showed that *S. haematobium* soluble egg antigen and the CCD marker bromelain were strong inhibitors of IgE binding to peanut extract.^33^ In a follow‐up study that used a synthetic glycan microarray to identify the specific glycan motifs associated with carbohydrate‐related IgE cross‐reactivity,^64^ *Schistosoma* infection was linked to IgE cross‐reactivity involving the core xylose glycan motif.^64^

Although further investigations are needed to examine how CCD‐specific IgE may inhibit allergic effector responses, Doenoff and colleagues have explored the role of IgG antibodies as blocking antibodies.^65^, ^66^ Through in vitro studies, rabbit anti‐schistosome IgG was found to cross‐react with allergens such as latex^65^ and peanut extract.^66^ The authors proposed that anti‐schistosome IgG antibodies may block IgE‐induced allergic responses and therefore prevent the manifestations of allergic disease.^66^

Generally, IgE to CCDs are not thought to be biologically active, but in recent years, responses to the mammalian carbohydrate epitope galactose‐α‐1,3‐galactose (α‐gal) have been of interest in the field of allergy since this epitope has been linked to two forms of anaphylaxis.^67^ The first being anaphylactic reactions following the infusion of the monoclonal antibody cetuximab among cancer patients undergoing therapy in the Southeastern United States^68^ and the second being delayed‐onset reactions hours after mammalian meat product consumption.^69^ Reactions to α‐gal have been documented from the Southeastern US, Central America, Australia and East Asia.^67^ A history of tick‐bite exposure has been shown to be strongly correlated with high levels of IgE to α‐gal.^70^ Specific tick species worldwide have been implicated in the induction of IgE to α‐gal such as the lone star tick (*Amblyomma americanum*) in the Southeastern United States,^64^ the castor bean tick (*Ixodes ricinus*) in Sweden,^71^ the Australian paralysis tick (*Ixodes holocyclus*) in Australia^72^ and the cattle tick (*Haemaphysalis longicornis*) in Japan.^64^ Although no direct link has been made between α‐gal and helminths in Africa, elevated levels of IgE to α‐gal have been seen in individuals from helminth‐endemic areas in Ghana,^64^ Kenya^73^ and Zimbabwe.^74^ In Ghana, α‐gal sensitization has been strongly linked to rural residence rather than current helminth infection,^64^ but further studies are needed to identify the specific factors associated with rural living that underlie this sensitization.

### 3.3 Cell‐mediated and Humoral Immune Mechanisms

Studies on immune mechanisms from population studies illustrate the role of helminths in protection from or susceptibility to allergy‐related outcomes. The anti‐inflammatory environment associated with helminths in the human host is characterized by elevated levels of the cytokines IL‐10 and TGF‐β, and general immune hyporesponsiveness.^18^ In addition, the importance of IL‐10 in helminth‐induced regulation of the allergic immune response has been shown in some population studies.^48^, ^75^ Over the past decade, a few studies conducted in African populations have sought to investigate the cellular immune mechanisms underlying helminth infections and the allergic immune response. For example, in an investigation combining a murine model and a human population study, Van der Vlugt et al demonstrated that *S. mansoni*‐mediated protection against experimental ova albumin‐induced allergic airway inflammation was dependent on IL‐10 producing B cells in mice.^76^ In the same publication, elevated levels of IL‐10 producing CD1d^hi^ B cells were observed in Gabonese children infected with *S. haematobium*. Based on these findings, the authors posited that IL‐10‐producing regulatory CD1d^hi^ B cells are induced in both humans and mice during chronic schistosomiasis. In mice, these IL‐10‐producing regulatory B cells have an important role in protection against experimental allergic inflammation.^76^

Other recent studies have also looked at how helminth‐induced cytokine profiles may influence allergic immune responses. For example, the investigation conducted on islands endemic for *S. mansoni* in Lake Victoria, Uganda, found that in a subset of participants, reported wheeze was negatively associated with *S. mansoni‐*specific cytokine responses.^77^ These observations are in line with the protective effects of helminth infection on allergy‐related outcomes.

When it comes to other immune mechanisms, a study among Ghanaian schoolchildren found that high expression of innate immune gene Toll‐like receptor 2 (TLR2) and suppressor of cytokine signalling (SOCS)‐3 messenger RNA (mRNA) was positively associated with SPT reactivity to house dust mite.^24^ At the same time, the expression levels of both TLR2 and SOCS‐3 were significantly lower in children infected with *S. haematobium*.^24^ Based on these observations, the authors hypothesized that systemic helminths such as *S. haematobium* may modify the development of the allergic immune response by modulating the expression levels of innate immune genes such as TLR2 and SOCS‐3.^24^ Although the specific mechanisms remain unknown, these findings are supported by other investigations that have observed diminished TLR expression and function linked to immune dysregulation in lymphatic filariasis; another disease caused by systemic helminths.^78^, ^79^

Allergen‐specific IgG4 is often considered as a marker of immune modulation while allergen‐specific IgE as a marker of allergic disease.^77^ The IgG4 to IgE ratio can be utilized to determine immune modulation over the allergic response. The aforementioned investigation conducted on the islands in Uganda also looked at the ratio of allergen‐specific IgG4 to IgE and found that house dust mite‐specific IgG4 to IgE ratio was significantly lower in those reporting wheeze (*P* = 0.032).^77^

In a study conducted in Zimbabwe, Rujeni and colleagues looked at the ratio of mite‐specific (Der p 1) IgE to IgG4 in a low *S. haematobium* transmission community as well as in a high transmission community.^31^ This mite‐specific IgE to IgG4 ratio provides an indicator of allergic response over immune modulation and the study found that *Schistosoma* infection intensity was negatively associated with Der p 1‐specific IgE to IgG4 ratio in the high transmission area.^31^ Following treatment for schistosomiasis, in the high transmission area there was no change in ratio of Der p 1‐specific IgE to IgG4 although the overall anti‐Der p 1 IgE responses declined.^80^ In the same province in Zimbabwe, antibody responses among pre‐school children between the ages of 3 and 5 years before and 6 weeks after treatment were investigated; treatment had no effect on Der p1‐specific IgE or IgG4 levels.^81^

The low‐affinity CD23 receptor is thought to be involved in the regulation of IgE synthesis.^82^ In the same Zimbabwean communities with low and high *S. haematobium* prevalence, Rujeni et al hypothesized that the levels of soluble CD23 would be negatively associated with allergen‐specific IgE titres as well as SPT wheal size in schistosome‐infected individuals.^83^ They observed that among 434 subjects, soluble CD23 levels were inversely associated with SPT reactivity to aeroallergens, specific IgE to Derp 1 and schistosome‐specific IgE.^83^ Overall, their findings suggest that soluble CD23 may play a role in the suppression of both schistosome‐specific and allergen‐specific IgE levels although further research is needed to elucidate regulatory mechanisms involving CD23.

Another immune mechanism that has been examined is the association between helminth infection and basophil cell suppression. A study in Uganda investigated the hypothesis that immunoregulatory responses that characterize helminth infections reduce the response of IgE effector cells, such as basophils, to IgE‐mediated activation resulting in suppression of responses to parasite and non‐parasite antigens.^37^ For this investigation, changes in specific and non‐specific histamine release in whole blood after anthelmintic treatment in schoolchildren from an area endemic for both *S. mansoni* and hookworm were examined.^37^ The study findings were that among children without detectable hookworm infection, a significant positive association was observed between circulating levels of house dust mite‐specific IgE and histamine release.^37^ However, this was not seen among children with hookworm infection, suggesting that this infection may have a suppressive effect on mite‐specific histamine release. Interestingly, a similar suppressive effect was not seen with *S. mansoni*.^37^

Studies conducted in Africa have observed a strong correlation between helminths and rural environments. For instance, studies that have reported an inverse association between helminths and allergy‐related outcomes were predominantly from rural areas.^24^, ^31^, ^37^ Since helminths are predominantly found in rural areas, observational studies may be faced with confounding from other factors in the rural environment that may not be fully adjusted for in statistical analysis. This makes it difficult to tease out whether helminths influence the risk of allergy‐related outcomes in their own right, or through interaction with other environmental factors in rural areas. This is exemplified by the two studies conducted in two different fishing communities along the shores of Lake Victoria in Uganda; both had high percentages of helminth infections but one showed an inverse association with wheeze^37^ while the other showed a positive association.^38^ Therefore, future studies should explore immune mechanisms related to rural environments aside from helminths that influence the pathogenesis of allergy‐related outcomes.

## 4 Conclusion

Studies from Africa investigating the association between helminths and allergy‐related outcomes have found a number of inconsistent observations, some showing inverse, positive or no associations. Since most of these studies are observational and conducted among school‐age children, establishing causation is impossible. However, these studies have illustrated how IgE cross‐reactivity between environmental or food allergens and helminth antigens has limited the diagnostic value of measuring IgE responses to whole allergen extracts in populations from helminth‐endemic areas. They also raise pertinent questions about the classification of asthma as “atopic”/”allergic” or not based on the measurement of allergen‐specific IgE to whole allergen extracts.

Although not consistent, studies on immune mechanisms have illustrated how helminth infections can induce immune regulatory responses that may protect against allergic immune responses. The observed lower burden of allergy‐related outcomes in rural compared to urban areas deserves further investigation. There is also evidence to suggest that the critical period worth investigating is early life. Therefore, studies in early life, either as anthelmintic trials during pregnancy and early childhood or as birth cohorts investigating a broad range of risk factors in both rural and urban areas, are needed. The current epidemiological transition in Africa offers an excellent opportunity to conduct such studies, so as to identify the primary factors driving the global rise in allergic disorders.

## DISCLOSURES

None.
